# Females and Exercise Capacity Impairment in Heart Failure: A Sex-Focused Analysis

**DOI:** 10.3390/jcdd12120494

**Published:** 2025-12-14

**Authors:** Ainhoa Lorenzo, Raúl Ramos-Polo, Laia Lorenzo-Esteller, Xinying Lin, Emma Barragan, Paula Aranda, Èlia Boixader, Foix Regull, Nerea Martín, Ariana Ollé, Marc Llagostera, Núria José-Bazán, Pedro Moliner, Cristina Enjuanes, Josep Comin-Colet

**Affiliations:** 1 Bio-Heart Cardiovascular Diseases Research Group, Bellvitge Biomedical Research Institute (IDIBELL), 08907 Barcelona, Spain; ainhoa.lorenzo.2001@gmail.com (A.L.); rramosp@bellvitgehospital.cat (R.R.-P.); llorenzo@bellvitgehospital.cat (L.L.-E.); xlinlinx8@alumnes.ub.edu (X.L.); emmabarragan.ub@gmail.com (E.B.); parandro8@alumnes.ub.edu (P.A.); eliaboixader@gmail.com (È.B.); fregulsa31@alumnes.ub.edu (F.R.); nereamar2000@gmail.com (N.M.); aollerov7@alumnes.ub.edu (A.O.); mllagosteram@bellvitgehospital.cat (M.L.); njose@bellvitgehospital.cat (N.J.-B.); pmoliner@bellvitgehospital.cat (P.M.); cenjuanes@bellvitgehospital.cat (C.E.); 2 School of Medicine, University of Barcelona, 08097 Barcelona, Spain; 3 Community Heart Failure Program, Cardiology Department, Bellvitge University Hospital, 08907 Barcelona, Spain; 4 Cardiology Department, Heart Institute, Bellvitge University Hospital, 08907 Barcelona, Spain; 5 Center for Biomedical Research Network for Cardiovascular Diseases (CIBERCV), 28029 Madrid, Spain; 6 Department of Clinical Sciences, School of Medicine, University of Barcelona, 08007 Barcelona, Spain

**Keywords:** heart failure, sex, quality of life, exercise tolerance, evidence-based healthcare

## Abstract

Heart failure (HF) is becoming increasingly common, especially in older females, and displays marked sex-related differences in pathophysiology, treatment, and outcomes. Submaximal exercise capacity (SEC), frequently measured by the six-minute walk test (6MWT), is an important marker of aerobic function, prognosis, and quality of life in HF. However, evidence regarding sex differences in SEC remains limited and inconsistent. This single-centre, prospective cohort study included 1069 patients with chronic HF enrolled between 2004 and 2014. SEC was assessed using the 6MWT, and extensive clinical and psychosocial data were collected. Multivariate models evaluated the independent association between sex and SEC. Results showed that females had significantly shorter 6MWT distances (155 ± 149 m) than males (265 ± 164 m; *p* < 0.001). Female sex was an independent predictor of impaired SEC in both unadjusted and adjusted analyses (odds ratios 2.226–3.609; *p* < 0.001). Additional determinants of reduced SEC included advanced age, higher NYHA class, elevated heart rate, diabetes, iron deficiency, dependence in activities of daily living, cognitive impairment, and depressive symptoms. These findings demonstrate that female sex is a strong, independent predictor of reduced functional capacity in chronic HF and emphasize the need for sex-specific strategies addressing both clinical and psychosocial factors to improve outcomes.

## 1. Introduction

Heart failure (HF) increases the risk of mortality and morbidity and affects patients’ self-perceived health status. The increasing incidence and corresponding rise in medical resource consumption pose a challenge to healthcare systems [1,2,3]. Recently, there has been a growing interest in identifying the existence of sex differences in the pathophysiology, clinical manifestations, and response to treatments in cardiovascular disease, particularly in patients with HF [4].

In this regard, hypertensive cardiopathy or valvular heart disease are more frequent HF aetiologies among females, whereas ischemic etiology is less frequent compared with males. Moreover, the hormonal profile in premenopausal females plays a protective effect, which contributes to a later age of onset of cardiovascular disease. Females tend to present different signs and are more prone to have a higher burden of comorbidities, which can impact their prognosis and may explain their worse outcomes, especially in the short term after HF hospitalization [5,6].

In recent years, the focus has been on defining sex differences in patient-reported outcome measures such as quality of life in patients with chronic HF [7,8]. In these studies, female sex was associated with worse quality of life (QoL) compared to males, despite adjustment for important prognostic determinants in HF. Impairments in QoL in patients with HF are mainly driven by limitations in the physical dimension of this patient-reported outcome measure (PROM), which, in turn, is widely determined by submaximal exercise capacity (SEC).

However, the impact of sex on SEC has not been adequately explored. Considering the role of SEC on self-perceived health status, there has been a growing interest in defining new interventions aimed to improve exercise capacity in these populations. SEC is an important measure of aerobic performance that can be easily assessed using the 6 min walk test. SEC correlates with prognosis and QoL in patients with HF and becomes an attractive measure to gauge physical limitations of patients and response after therapeutic interventions. Also, it is useful as a risk stratification tool to guide decision making [9]. Despite the importance of SEC as a prognostic marker, little research has been conducted to address the existence of sex differences in this functional parameter. Given the limitations mentioned above, our study was designed to investigate whether differences in SEC exist between males and females with chronic HF and to explore how clinical and psychosocial factors may influence these differences.

## 2. Materials and Methods

### 2.1. Study Design and Patient Population

The Definition of the neuro-hormonal Activation, Myocardial function, genOmic expression, and CLinical outcomes in hEart failure patients (DAMOCLES) study was a single-centre, observational, prospective cohort study of 1236 consecutive patients diagnosed with CHF recruited between January 2004 and January 2013. This study was designed and reported following the STROBE guidelines for observational cohort studies. A completed STROBE checklist is provided in the Supplementary Material.

The methodology of the DAMOCLES study has been published previously [8,10,11]. Briefly, for inclusion, patients must have been diagnosed with HF according to the European Society of Cardiology diagnostic criteria, had at least one recent acute decompensation of HF requiring intravenous diuretic therapy (either hospitalized or in the day care hospital), and had to be in a stable condition at the time of study entry. Exclusion criteria were significant primary valvular disease, clinical signs of fluid overload, pericardial disease, restrictive cardiomyopathy, hypertrophic cardiomyopathy, hemoglobin (Hb) levels < 8.5 g/dL, active malignancy, and chronic liver disease. Only patients with missing baseline information were excluded.

The study was approved by the local committee of ethics for clinical research and was conducted in accordance with the principles of the Declaration of Helsinki. All patients gave written informed consent before study entry.

### 2.2. Baseline Assessment

A detailed baseline evaluation was performed for all participants at study entry. This included the collection of information about demographic characteristics and exhaustive medical history to gather clinical- and disease-related factors such as NYHA functional class, co-morbidities, laboratory information, medical treatments, and the most recent left ventricular ejection fraction (LVEF).

### 2.3. Psychosocial Evaluation

To fully characterize patients in their psychosocial dimension, several validated instruments were administered.

Cognitive function was evaluated by means of the administration of the Short Portable Mental State Questionnaire and the Mini-Mental State Examination questionnaire. Cognitive impairment was defined as abnormal scoring in any of these two questionnaires (MMSE < 24 or ≥3 mistakes in the SPMSQ) [12,13].

Dependency to perform basic activities of daily living (ADLs) was evaluated by the Barthel Index. The scores range from 0 (total dependence) to 100 (independence). Dependency to perform instrumental ADLs was evaluated using the Lawton and Brody scale. The scores range from 0 to 8 (total independency), from 8 to 20 (mild dependency), and more than 20 (moderate–severe dependency) [14].

Finally, affective status was evaluated using the 15-item Geriatric Depression Scale. In this scale, scores range from 0 to 15. Abnormal affective status, defined by the presence of depressive symptoms, was determined using a cut-off point ≥4 points in the GDS-15 score [15,16].

According to these tests, fragility was defined as the presence of cognitive impairment, moderate–severe dependency for instrumental activities, dependency for basic ADLs, or the presence of depressive symptoms.

### 2.4. Submaximal Exercise Capacity Evaluation

The 6MWT is a widely used clinical assessment tool to evaluate exercise capacity and functional status [10]. The methodology of this test has been previously published [17,18]. The 6MWT measures the distance a patient can walk in six minutes on a flat surface, assessing functional capacity, disease progression, treatment response, and prognosis, with pre- and post-test parameters recorded. Impaired submaximal exercise capacity included patients walking less than 300 m or who were unable to complete the 6MWT.

### 2.5. Statistical Analyses

Using the baseline data from the DAMOCLES cohort, a cross-sectional analysis was performed. Demographic and clinical characteristics, laboratory tests results, and submaximal exercise capacity were summarized using basic descriptive statistics, both overall and categorized by sex. For categorical variables, number and percentage were reported. For continuous variables, mean (standard deviation) or median (inter-quartile range) were used, depending on the distribution of the variables. χ2, Student’s T, and non-parametric tests were used to compare characteristics across strata.

To define the relative contribution of sex and its interplay with disease-related factors and psychosocial factors on SEC, we constructed several multivariable models. First, we developed multivariate regressions analyses using both binary logistic and linear regression methods to explore the contributions of disease-related or psychosocial determinants on SEC separately (split models). Second, we developed multivariate regressions analyses using the same methods to explore the joint contributions of disease-related and psychosocial determinants of SEC (joint models). All multivariable models were developed using backward methods. Since a covered distance of 0 m was imputed to patients who were unable to walk until completion of the 6MWT, a specific analysis was performed including only those who were able to complete the test (sensitive analysis). Finally, the effect of the interaction between sex and psycho-functional aspects of the disease on SEC were evaluated using dedicated models; we performed adjusted General Linear Models (GLMs) to assess the association between sex and submaximal exercise capacity according to psycho-functional status. GLMs were fitted with 6MWT distance and interaction terms between sex and each psycho-functional variable.

All statistical tests and confidence intervals (CIs) were constructed with a type I error alpha level of 5%, with no adjustments for multiplicity. *p* values below 0.05 were considered statistically significant. All analyses were performed using SPSS software (Version 27) and R software (R 4.5.2).

## 3. Results

Our cohort included 1069 patients, comprising 457 females (43%), the mean age was 72 years, and the mean LVEF was 45%. The general and sex-specific psychosocial aspects, as well as the disease-related characteristics of the patients, are shown in Table 1.

Females were older (75 vs. 71 years old, *p*-value < 0.001) and more frequently hypertensive than males. They also had lower creatinine and hemoglobin levels and had more frequent iron deficiency. Although preserved LVEFs were more prevalent, the female functional class was worse compared to males. In contrast, the prevalence of ischaemic heart disease as well as a previous episode of myocardial infarction was more frequent in males.

A more advanced frailty profile was observed among females, as illustrated by a higher rate of dependence for basic and instrumental activities, higher frequency of cognitive impairment, and more depressive symptoms (all *p*-values < 0.05). As mentioned, these psycho-functional factors are summarized in the variable fragility criteria: 346 (81.2%) females vs. 289 (53.9%) males met the criteria (*p*-value < 0.001).

**Figure 1:**
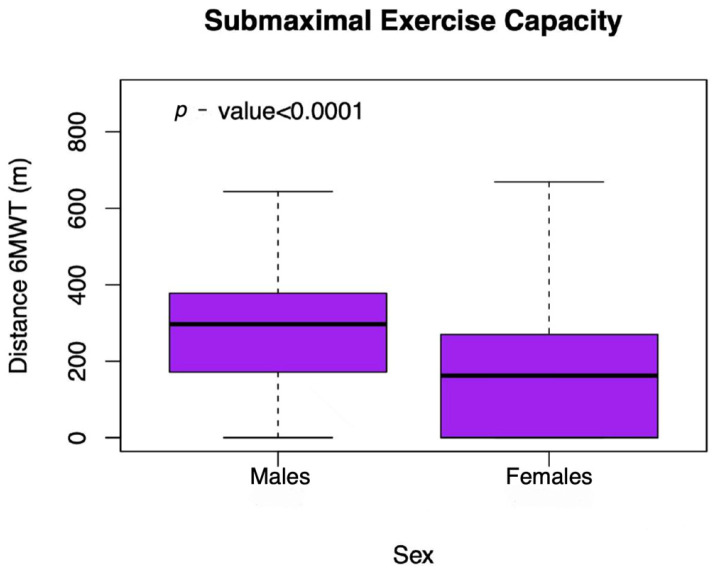
Box plots showing the unadjusted association between sex and submaximal exercise capacity measured using the 6 min walking test where distance is expressed in metres.

As presented in Figure 1, females performed a significantly shorter distance in 6MWT (155 ± 149 265 ± 164 m; *p*-value < 0.001). They were more likely to be unable to perform the 6MWT (40.5% vs. 20.1%, *p*-value < 0.001) and to cover less than 300 m (40.5% vs. 19.0%, *p*-value < 0.001) in comparison to males (Figures S1 and S2). Additionally, females walked shorter distances after excluding patients who were unable to perform the 6MWT (261 ± 100 vs. 332 ± 108, *p*-value < 0.001) and presented impaired SEC more frequently (68% vs. 40.1%, *p*-value < 0.001).

### 3.1. Determinants of Submaximal Exercise Capacity in Unadjusted Analyses

Female sex was associated with a shorter distance walked in the 6MWT, indicating lower SEC in univariate linear regression (standardized coefficient β = −0.307; *p*-value < 0.001) (Table S1). Regarding psycho-functional factors, dependency for ADLs (standardized coefficient β = −0.407; *p*-value < 0.001), moderate or severe dependency for instrumental activities (standardized coefficient β = −0.343; *p*-value < 0.001), cognitive impairment (standardized coefficient β = −0.245; *p*-value < 0.001), and depressive symptoms (standardized coefficient β = −0.119; *p*-value = 0.002) were associated with poor functional capacity. Other disease-related variables (age, LVEF, NYHA functional class, iron deficiency, and NT-proBNP, among others) were associated with an impaired SEC in unadjusted analysis.

### 3.2. The Role of Sex in Models Adjusted for Clinical Determinants

**Figure 2:**
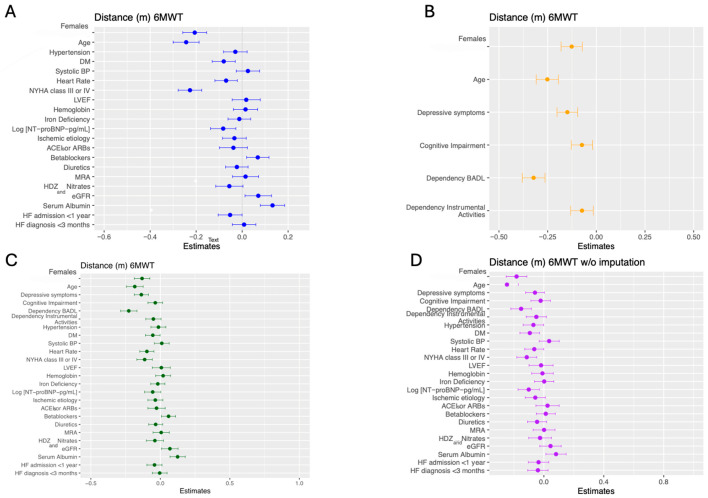
Standardized β coefficients and standard errors obtained using multivariate linear regression analysis evaluating the association of sex, disease-related (clinical), and psycho-functional factors with exercise capacity defined as distance walked in the 6 min walk test. Multivariate analyses are presented in 2 models: a split model (separately for disease-related and psycho-functional factors (**A**,**B**) and a joint model (disease-related and psycho-functional factors combined in the same predictive model (**C**,**D**).

In the multivariate binary logistic regression analyses adjusted for disease-related determinants (Table 2), female sex was associated with worse submaximal exercise capacity (OR: 3.609; CI: 2.462–5.291; *p*-value < 0.001). This association was also shown by multivariable linear regression models adjusted for disease-related factors (shown in Table S1 and Figure 2).

### 3.3. The Role of Sex in Models Adjusted for Psycho-Functional Determinants

In the multivariate binary logistic regression analyses adjusted for psycho-functional determinants (Table 2), female sex remained an independent predictor of worse performance in the 6MWT (OR: 2.226; CI: 1.537–3.224; *p*-value < 0.001). Multivariable linear regression models adjusted for disease-related factors (Table S1, Figure 2) showed the same results. In these models, dependency for ADLs, dependency for instrumental activities, and cognitive impairment were also associated with SEC.

### 3.4. The Role of Sex in Comprehensive Adjusted Models Including Clinical and Psychosocial Determinants

Multivariate binary logistic regression analyses (Table 2) showed female sex was an independent predictor of worse SEC (OR: 2.681; CI: 1.709–4.204; *p*-value < 0.001). Female sex showed the same association with a worse performance in the 6MWT (standardized coefficient β = −0.128; *p*-value < 0.001) (Table S1, Figure 2).

### 3.5. The Role of Sex in Sensitivity Analyses

After excluding patients who were unable to walk and perform the 6MWT, joint models showed female sex was substantially associated with a lower capacity for exercise (standardized β: −0.188; *p*-value < 0.001) (Figure 2). Additionally, we incorporated fragility as an all-encompassing indicator of psycho-functional constraints (Figure S3), obtaining standardized β coefficients for the split model (β: −0.166; *p*-value < 0.001) and joint model (β: −0.158; *p*-value < 0.001).

**Figure 3:**
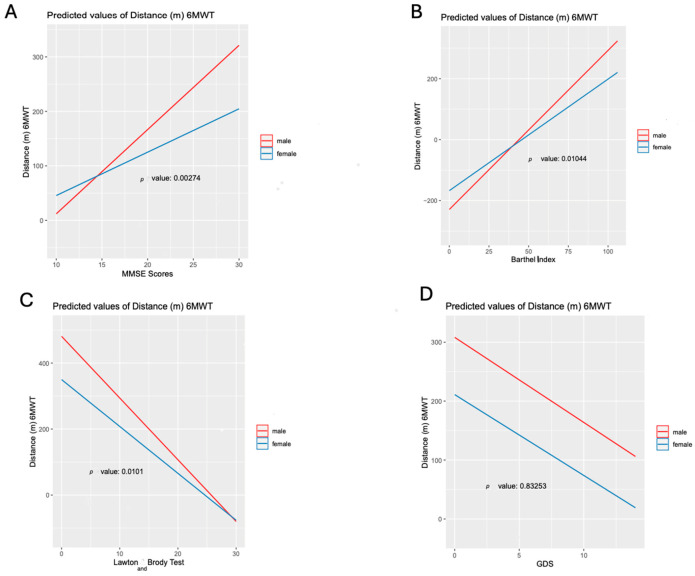
Graphical exploration of the effect of interaction between: (**A**) sex and MMSE scores; (**B**) sex and Barthel index; (**C**) sex and Lawton and Brody test; (**D**) sex and GDS score; interaction effects graphs using adjusted GLM models.

Finally, we developed GLMs to assess whether the association between sex and submaximal exercise capacity varied according to psycho-functional status; we fitted adjusted GLMs with a single continuous dependent variable (6MWT distance) and interaction terms between sex and each psycho-functional variable (MMSE score, Barthel Index, Lawton and Brody scale, and GDS score) in Figure 3. These analyses showed a significant interaction between SEC and cognitive impairment (measured by the MMSE score), and dependency for ADLs and instrumental activities (measured by the scores in the Barthel index and Lawton and Brody test, respectively) (all *p*-values < 0.05). No significant interaction on the distance covered in the 6MWT was found between sex and the GDS score as a measure of affective status (*p*-value = 0.832).

## 4. Discussion

In this single-centre prospective cohort study in patients with HF, females demonstrated worse SEC. Significant determinants of SEC included clinical factors (age, NYHA class, heart rate, diabetes, and iron deficiency) and psychosocial factors (dependency for activities of daily living, cognitive impairment, and depressive symptoms). Female sex was an independent predictor of worse SEC in multivariable analyses. This research highlights how psychosocial factors modulate SEC, with an additive effect over clinical parameters. Notably, this is the first study showing significant differences in SEC between sexes measured by 6MWT. Thus, sex is proposed as the main predictor of functional capacity (Graphical Abstract).

Previous studies have proposed an association between sex and exercise capacity [19,20]. In contrast, other research [21,22] showed mixed results in the similarity of exercise capacity between sexes. These works were not conclusive mainly due to reduced sample size, an underrepresentation of females, the exclusive evaluation of patients with HFpEF (Heart Failure with Preserved Ejection Fraction), and the measurement of peak exercise capacity but not SEC. The specific interaction between sex and psychosocial or additional functional factors in SEC measured by 6MWT has never been explored.

The 6MWT is an easily performed test that requires minimal resources. Although cardiopulmonary exercise testing generates a large amount of physiological information, 6MWT is less physically demanding and highly reproducible, making it more feasible for older HF patients. Furthermore, 6MWT correlates with disease severity, prognosis, and quality of life [9]. However, it misses peak exercise performance data and lacks specificity, as non-cardiac factors such as age and motivation can influence the results.

According to our data, sex determines SEC independently of other important clinical and psychosocial factors related to frailty and physical limitation. Biological differences, such as post-menopausal hormonal changes, can exacerbate age-related declines in physical capacity [23,24,25,26]. Males typically have greater height and muscle mass, heart size, lung function, and cardiovascular response, all of which contribute to better exercise performance [27]. Testosterone plays a significant role in muscle mass, strength, and blood flow during exercise, enhancing metabolic efficiency [28]. Additionally, iron deficiency, which is more common in females (62.1% vs. 55.3%, *p* = 0.029), is linked to reduced aerobic capacity and physical performance [29,30].

In our cohort, females were slightly older than males (75 vs. 71 years, *p* < 0.001). Thus, females could over-express age-related factors as loss of muscle mass, decreased cardiopulmonary function, and bone density. Moreover, comorbidities such as arthritis and osteoporosis are more frequent in females. Those conditions contribute to reduced mobility, joint pain, and musculoskeletal impairments, directly affecting exercise capacity [31,32].

Beyond biological factors, the psychosocial sphere directly affects exercise capacity. Females are more likely to report higher pain levels and greater sensitivity to pain due to conditions such as musculoskeletal disorders. They also exhibit higher levels of stress and anxiety, which can further impair their physical performance [33].

Finally, suboptimal healthcare management of female patients is also a relevant factor conditioning clinical course and symptom expression of the diseases. Healthcare facilities may not offer specialized services or programmes tailored to the unique needs of females with HF. There is a sex bias in healthcare, as females may be less likely to be referred for advanced diagnostic testing or specialized treatments, leading to delays in diagnosis [34,35]. Furthermore, females may be less likely to recognize signs and symptoms of HF and therefore do not seek medical attention promptly enough. This could be explained by cultural norms that prioritize caregiving responsibilities and the health needs of third parties over their own. Lastly, there is a notable underrepresentation of females in HF studies [20]. Historically, clinical research in cardiovascular diseases has disproportionately included male participants, leading to a lack of understanding of how the disease manifests and progresses in females [36].

Our research shows clear sex differences in SEC measured by 6MWT. Females displayed worse exercise capacity even considering other clinical and psychosocial determinants. Future research is needed to explore other disease-related and psychosocial variables that could potentially explain females’ worse functional capacity and quality of life.

## 5. Conclusions

In this cohort, we concluded that female sex is a determinant of worse submaximal exercise capacity in patients with heart failure, regardless of prognostic clinical-, psychosocial-, and frailty-related factors. Future studies are needed to characterize the causes of this gap in submaximal exercise capacity based on sex and to design therapeutic strategies to mitigate it.

### Limitations

This study has several inherent limitations related to its cross-sectional design. First, it does not capture longitudinal changes in health status or dynamic relationships with clinical variables over time. Second, as an observational study, it cannot establish causal inferences. Third, being a single-centre study limits the generalizability of the findings to broader HF populations, although our cohort is comparable to recent HFrEF (Heart Failure with reduced Ejection Fraction) studies.

The 6 min walk test (6MWT) has limited sensitivity due to variability from motivation and psychological factors. However, it remains a simple, accessible, and cost-effective tool for assessing submaximal exercise capacity in HF patients. Compared to cardiopulmonary exercise testing (CPET), which provides more detailed data on VO_2_ max and ventilatory efficiency, the 6MWT is a more feasible alternative in most clinical settings, as CPET requires specialized equipment and trained personnel.

In this study, partially completed 6MWT distances were not recorded for participants who were unable to finish the test. Although intermediate distances would have provided a more precise reflection of residual ambulatory capacity, the dataset only captured whether the test was completed or not. Consequently, assigning 0 m to incomplete tests was the only feasible and internally consistent approach, avoiding non-standard assumptions or post hoc estimation of unrecorded values. This limitation should be acknowledged, as it reduces the granularity of functional assessment and may underestimate the true ambulatory ability of participants who attempted but did not complete the 6MWT.

## Figures and Tables

**Table 1:** Baseline characteristics of the study cohort, both overall and according to sex.

	Whole Cohort (n = 1069)	Females (n = 457)	Males (n = 612)	*p*-Value
Demographic and Clinical Factors				
Age (years)	72 ± 11	75 ± 10	71± 12	<0.001
Systolic blood pressure (mmHg)	124 ± 22	124 ± 21	124 ± 22	0.516
Heart rate (bpm)	734 ± 14	75 ± 15	73 ± 14	0.063
NYHA functional class, n (%)				<0.001
I	140 (13%)	30 (7%)	110 (18%)	
II	497 (47%)	199 (44%)	298 (49%)	
III	351 (33%)	183 (40%)	168 (28%)	
IV	76 (7%)	43 (9%)	33 (5%)	
HF hospitalisation previous year, n (%)	883 (83%)	391 (86%)	492 (81%)	0.031
HF diagnosis > 1 year, n (%)	406 (38%)	164 (36%)	242 (40%)	0.215
Left ventricular ejection fraction (%)	45 ± 17	50 ± 17	40 ± 16	<0.001
HFpEF, n (%)	636 (60%)	204 (45%)	432 (71%)	<0.001
Ischaemic etiology of HF, n (%)	406 (38%)	116 (25%)	290 (47%)	<0.001
Comorbidities				
Hypertension, n (%)	859 (80%)	387 (85%)	472 (77%)	0.002
Diabetes mellitus, n (%)	494 (46%)	217 (48%)	277 (45%)	0.486
Previous MI, n (%)	276 (26%)	64 (14%)	212 (35%)	<0.001
CKD, n (%)	585 (55%)	306 (67%)	279 (50%)	<0.001
Anemia, n (%)	518 (49%)	234 (51%)	284 (47%)	0.120
Iron deficiency, n (%)	610 (57%)	278 (62%)	332 (55%)	0.029 *
Treatments (%)				
ACEIs or ARBs	780 (73%)	333 (73%)	447 (73%)	0.992
Beta-blockers	941 (88%)	396 (86%)	545 (89%)	0.232
MRA	398 (37%)	137 (30%)	261 (42%)	<0.001
Diuretics	970 (90%)	427 (93%)	543 (88%)	0.009
Antiplatelet or anticoagulant therapy	880 (82%)	362 (79%)	518 (84%)	0.021
Laboratory				
Hemoglobin (g/dL)	12.7 ± 2.3	12.1± 1.7	13.1± 2.6	<0.001
Creatinine (mg/dL)	1.33 ± 0.60	1.24 ± 0.55	1.40± 0.63	<0.001
NT-proBNP (pg/mL)	1583 [685–3766]	1657 [752–3890]	1516 [620–369]	0.422
Serum albumin (g/dL)	3.9 ± 0.49	3.8 ± 0.5	3.9 ± 0.5	0.003
Psycho-functional Factors				
Barthel index (points)	92 ± 16	88 ± 18	94 ± 14	<0.001
Dependency for basic ADLs, n (%)	366 (34%)	216 (54%)	150 (28%)	<0.001
Moderate or severe dependency for basic ADLs, n (%)	264 (24%)	162 (40%)	102 (19%)	<0.001
Lawton and Brody test (points)	13 ± 5	14 ± 6	12 ± 5	<0.001
Dependency instrumental activities, n (%)				<0.001
No dependency	250 (23%)	73 (17%)	177 (31%)	
Mild dependency	641 (60%)	293 (70%)	348 (62%)	
Moderate or severe dependency	90 (8%)	55 (13%)	35 (6%)	
Cognitive impairment, n (%)	344 (32%)	209 (46%)	135 (22%)	<0.001
Score in the Geriatric Depression Scale (GDS), points	3.6 ± 3.0	4.1 ± 3.0	3.2 ± 2.9	<0.001
Depressive symptoms, n (%)	298 (27%)	161 (41%)	137 (26%)	<0.001
Fragility criteria +	635 (59%)	346 (81%)	289 (54%)	<0.001

* MI: myocardial infarction. CKD: chronic kidney disease. ACEIs: angiotensin-converting enzyme inhibitors. ARBs: angiotensin receptor blockers. MRA: mineral-corticoid receptor antagonists.

**Table 2:** Univariate and multivariate binary logistic regression analyses (backward method) evaluating the association of sex, disease-related (clinical), and psycho-functional factors with impaired submaximal exercise capacity defined as distance walked in the 6 min walking test < 300 m. Multivariate analyses are presented in 2 models: a split model (separately for disease-related and psycho-functional factors) and joint model (disease-related and psycho-functional factors combined in the same predictive model).

	UNIVARIATE			MULTIVARIATE (SPLIT)			MULTIVARIATE (JOINT)		
	OR	95% IC	*p*-Value	OR	95% IC	*p*-Value	OR	95% IC	*p*-Value
Disease-related and demographic determinants									
Females, women vs. males	3.906	2.946–5.180	<0.001	3.609	2.462–5.291	<0.001	2.681	1.709–4.204	<0.001
Age	1.095	1.080–1.110	<0.001	1.072	1.053–1.092	<0.001	1.056	1.034–1.078	<0.001
Systolic blood pressure, mmHg	0.997	0.991–1.003	0.315	0.997	0.989–1.005	0.506	0.999	0.990–1.008	0.846
Heart rate, bpm	1.015	1.006–1.024	0.001	1.008	0.996–1.020	0.174	1.016	1.002–1.030	0.028
NYHA functional class, III–IV vs. I–II	5.464	4.016–7.434	<0.001	3.050	2.094–4.441	<0.001	1.765	1.137–2.740	0.011
HF hospitalization previous year	3.348	2.415–4.641	<0.001	1.710	1.088–2.688	0.020	1.497	0.903–2.481	0.118
HF diagnosis <3 months (recent)	1.436	1.116–1.848	0.005	1.274	0.882–1.840	0.197	1.343	0.878–2.054	0.174
Left ventricular ejection fraction, 1%	1.018	1.011–1.026	<0.001	0.993	0.980–1.006	0.289	0.995	0.979–1.010	0.499
Ischaemic aetiology	1.219	0.939–1.581	0.136	1.342	0.917–1.965	0.130	1.432	0.925–2.217	0.107
Hypertension	2.996	2.199–4.082	<0.001	1.668	1.079–2.577	0.021	1.530	0.918–2.550	0.102
Diabetes mellitus	1.966	1.519–2.545	<0.001	1.712	1.206–2.430	0.003	1.435	0.960–2.144	0.078
Iron deficiency	1.867	1.445–2.413	<0.001	1.035	0.730–1.467	0.849	1.010	0.678–1.506	0.960
Laboratory and treatment									
ACEIs or ARBs, yes vs. no	0.459	0.337–0.625	<0.001	0.917	0.559–1.504	0.731	0.747	0.424–1.316	0.313
Beta-blockers, yes vs. no	0.401	0.254–0.632	<0.001	0.490	0.272–0.881	0.017*	0.698	0.369–1.320	0.268
MRA, yes vs. no	0.595	0.460–0.769	<0.001	1.129	0.750–1.700	0.562	1.129	0.708–1.802	0.609
Diuretics, yes vs. no	2.847	1.867–4.340	<0.001	1.432	0.784–2.615	0.242	1.622	0.829–3.174	0.157
Hydralazine and nitrate, yes vs. no	2.294	1.628–3.233	<0.001	1.176	0.694–1.992	0.546	0.981	0.539–1.785	0.949
Hemoglobin, g/dL	0.729	0.678–0.784	<0.001	0.973	0.899–1.052	0.490	0.968	0.879–1.065	0.502
logNT-proBNP, log[1pg/mL]	3.064	2.391–3.925	<0.001	1.407	0.999–1.980	0.050	1.294	0.871–1.923	0.202
eGFR, (ml/min/1.73 m^2^)	0.974	0.968–0.979	<0.001	0.992	0.984–1.000	0.049	0.993	0.984–1.001	0.096
Serum albumin, g/dL	0.204	0.148–0.281	<0.001	0.476	0.326–0.694	<0.001	0.459	0.294–0.716	<0.001
Adjusted R^2^ for each model	-	-	-	0.415					
Psychosocialand demographic determinants									
Sex, women vs. men	-	-	-	2.226	1.537–3.224	<0.001	-		-
Age, 1 year	-	-	-	1.066	1.048–1.084	<0.001	-		-
Moderate or severe dependency for basic ADLs, yes vs. no	12.068	8.021–18.156	<0.001	6.159	3.904–9.716	<0.001	4.837	2.877–8.130	<0.001
Moderate or severe dependency instrumental activities, yes vs. no	5.043	3.713–6.849	<0.001	1.806	1.229–2.655	0.003	1.660	1.079–2.555	0.021
Cognitive impairment, yes vs. no	4.036	2.930–5.558	<0.001	1.675	1.106–2.536	0.015	1.373	0.870–2.166	0.173
Depressive symptoms, yes vs. no	2.177	1.594–2.973	<0.001	1.409	0.948–2.095	0.067	1.310	0.839–2.045	0.235
Adjusted R^2^ for each model	-			0.318			0.483		

OR: odds ratio. IC: confidence interval. ACEIs: angiotensin-converting enzyme inhibitors. ARBs: angiotensin receptor blockers. MRA: mineral-corticoid receptor antagonists.

## Data Availability

The original contributions presented in this study are included in the article/Supplementary Materials. Further inquiries can be directed to the corresponding author

## Supplementary Materials

The following supporting information can be downloaded at: https://doi.org/10.5281/zenodo.15776463 (accesses on 1 October 2025), Table S1: Univariate and multivariate linear regressions analyses (backward method) evaluating the association of sex, disease-related (clinical), and psycho-functional factors with submaximal exercise capacity defined as distance walked in the 6 min walking test. Multivariate analyses are presented in 2 models: a split model (separately for disease-related and psycho-functional factors) and joint model (disease-related and psycho-functional factors combined in the same predictive model); Figure S1: Proportions of patients describing the performance in the 6MWT according to sex; Figure S2: Proportions of patients with impairment in exercise capacity defined as 6MWT distance <300 m or unable to perform the 6MWT due to functional limitation stratified by sex; Figure S3: Standardized β coefficients and standard errors obtained using multivariate linear regression analysis evaluating the association of sex, disease-related (clinical), and psycho-functional factors with impaired submaximal exercise capacity, using “fragility” as a comprehensive measure of psycho-functional limitations.

## Abbreviations

The following abbreviations are used in this manuscript:

ADLs	Activities of Daily Living
CHF	Chronic Heart Failure
DAMOCLES	Definition of the neuro-hormonal Activation, Myocardial function, genOmic expression, and CLinical outcomes in hEart failure patients
ESC	European Society of Cardiology
GDS	15-item Geriatric Depression Scale
GLM	Generalized Linear Model
Hb	Hemoglobin
HF	Heart Failure
HFpEF	Heart Failure with preserved Ejection Fraction
HFrEF	Heart Failure with reduced Ejection Fraction
LVEF	Left Ventricular Ejection Fraction
MMSE	Mini-Mental State Examination questionnaire
MI	Myocardial Infarction
NYHA	New York Heart Association
PROMs	Patient-Reported Outcome Measures
QoL	Quality of Life
SEC	Submaximal Exercise Capacity
SPMSQ	Short Portable Mental State Questionnaire
6MWT	6-Minute Walk Test
